# An ulcerative and papular eruption in the groin and scalp

**DOI:** 10.1016/j.jdcr.2026.06.069

**Published:** 2026-07-16

**Authors:** Tyler C. Chinyere, David Baltazar

**Affiliations:** a University of Arizona College of Medicine-Phoenix, Phoenix, Arizona; b Medical Dermatology Specialists Phoenix, Phoenix, Arizona

**Keywords:** adult, cutaneous langerhans cell histiocytosis, langerhans cell histiocytosis

## Case presentation

**Fig 1:**
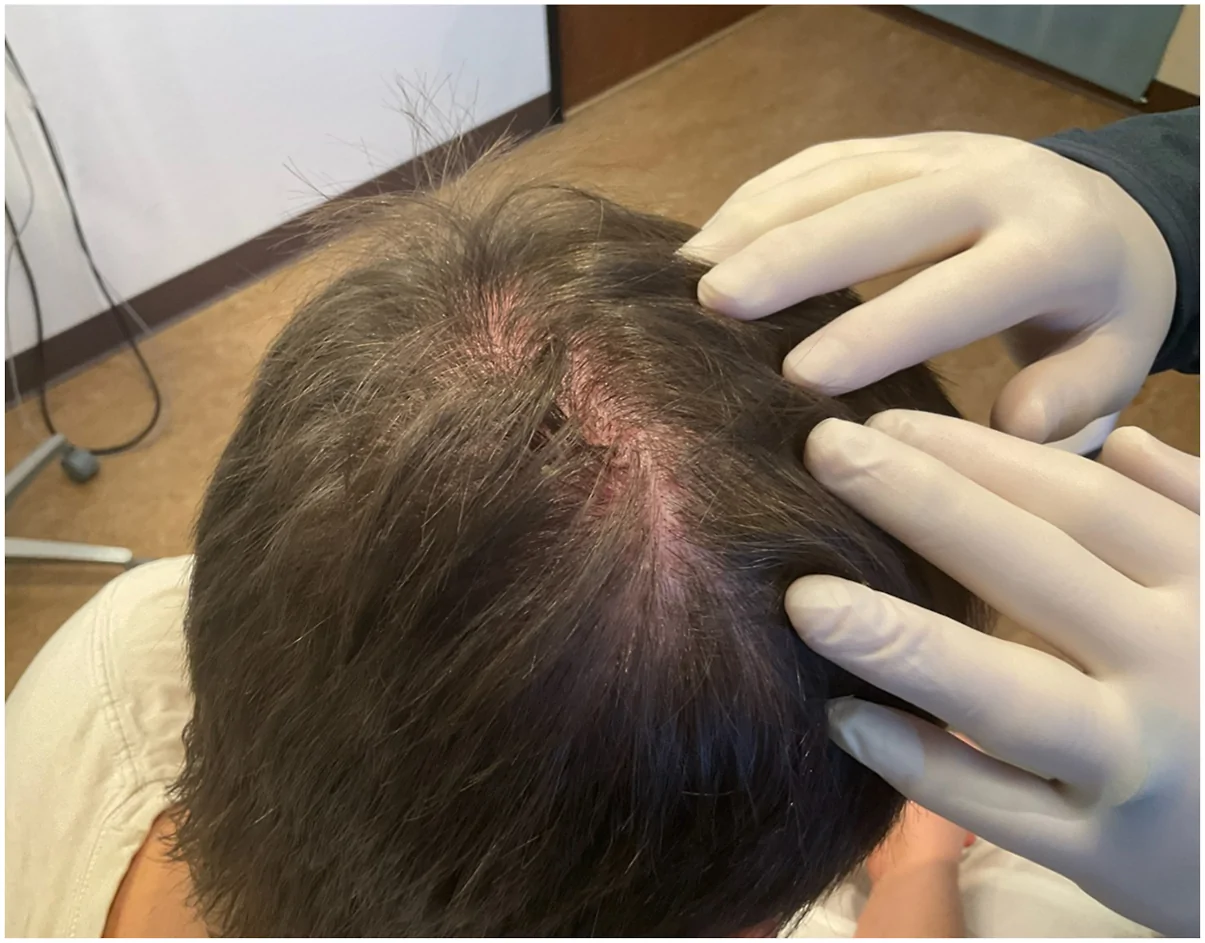
Scalp papules with generalized erythema.

**Fig 2:**
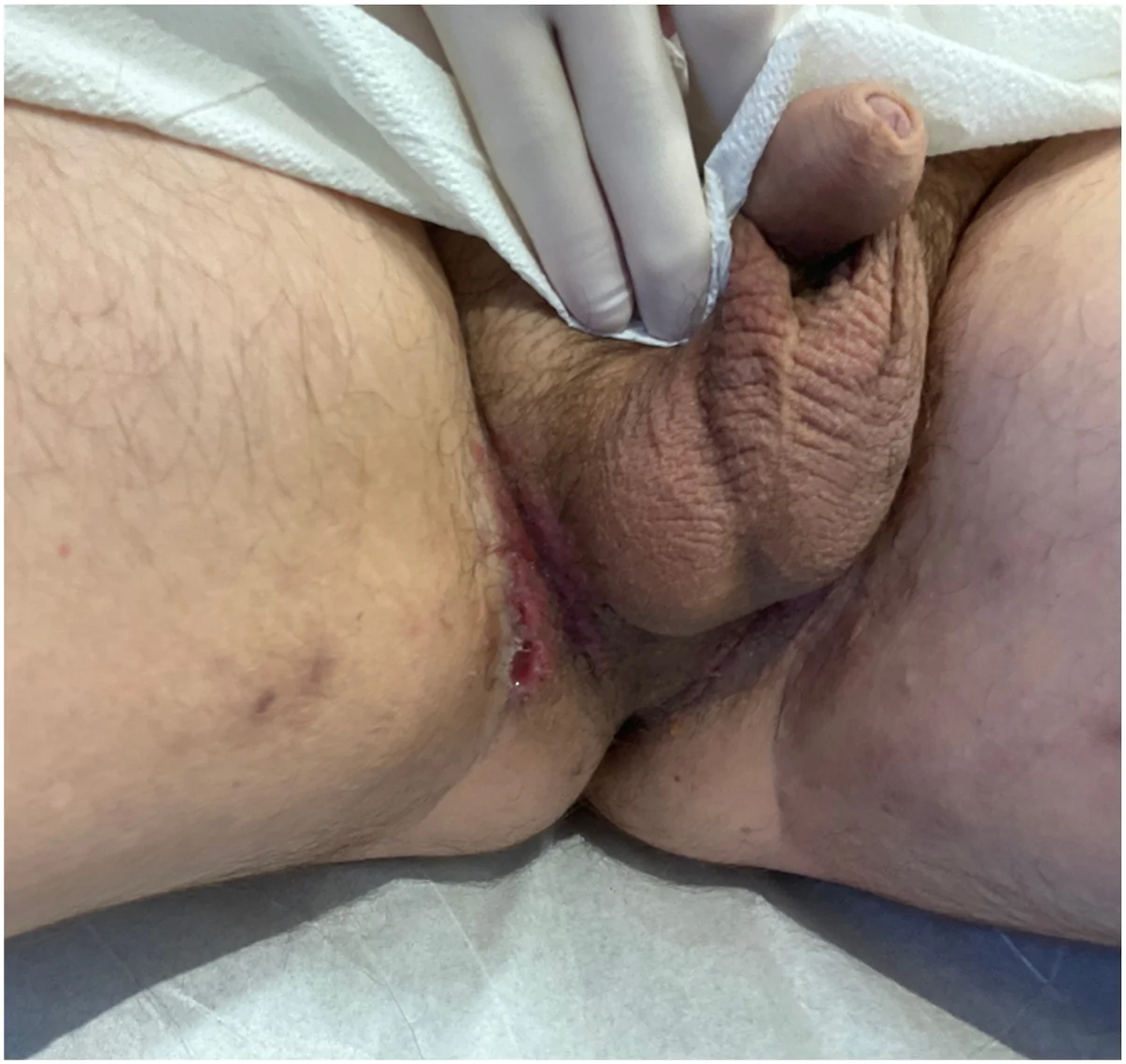
Uclerated plaques with satellite papules on the right inguinal region.

**Fig 3:**
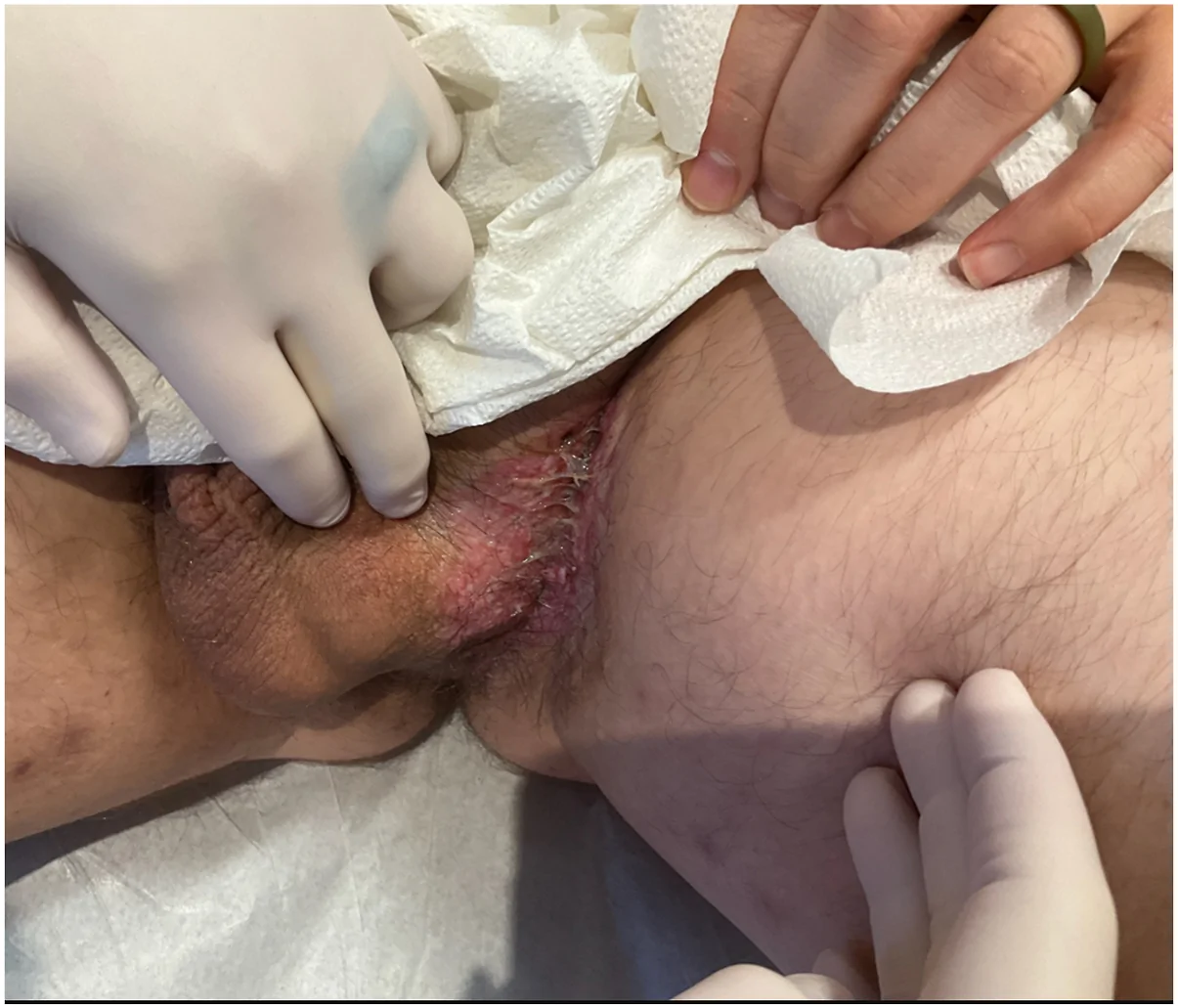
Uclerated plaques with satellite papules on the left inguinal region.

A 33-year-old male presented to the dermatology clinic with a 2-year history of a persistent worsening groin rash, along with a recent onset of painful scalp lesions over the past 6 months (Fig 1, Fig 2, Fig 3). Initially, the groin eruption began as a small papule on the left side and gradually evolved into a painful, ulcerated lesion. Within the last 6 months, the eruption extended to the right inguinal region. Concurrently, he developed tender bumps on his scalp accompanied by diffuse irritation. One year prior, at another office, he completed two 30-day courses of doxycycline with no improvement. Physical examination revealed erythematous to reddish-brown ulcerated plaques with satellite papules located in the inguinal folds bilaterally. Examination of the scalp showed multiple yellow to brown papules on a background of generalized erythema. His past medical history was significant for a pituitary tumor diagnosed in 2016, for which he had been receiving vasopressin replacement due to central diabetes insipidus. Histopathologic examination of the biopsy revealed a dense dermal infiltrate composed of large, pale-staining histiocytoid cells with irregular, reniform nuclei, and abundant eosinophilic cytoplasm. Scattered eosinophils and lymphocytes were admixed within the infiltrate, while the epidermis was largely spared. Immunohistochemical staining demonstrated strong positivity for CD1a, CD207, and S100. CD68 staining and BRAF V600E mutation analysis were negative.

**Question: Based on the clinical presentation above, what is the diagnosis?**

**A.** Cutaneous Crohn disease
**B.** Hidradenitis suppurativa
**C.** Leukemia cutis
**D.** Langerhans cell histiocytosis
**E.** Indeterminate cell histiocytosis

## Discussion

Langerhans cell histiocytosis (LCH) is a rare neoplastic disorder of Langerhans cells (LC), an antigen-presenting cell commonly located in the stratum spinosum of the epidermis.^1^ LCH can be classified into single-system Langerhans cell histiocytosis or multisystem LCH. In adults, multisystem LCH is the most prevalent form and commonly presents with central diabetes insipidus.^2^ The diabetes insipidus in our case was stemming from a radiographically stable pituitary tumor that was being monitored annually by neurosurgery for 8 years. The pituitary tumor was initially thought to represent a benign, isolated process rather than an infiltrative condition such as LCH. This demonstrates that LCH can be a chronic condition with new symptoms presenting over time. Other manifestations of LCH that should prompt further investigation include respiratory symptoms, bone lesions and fractures, and jaundice. Compared to pediatric cases, adult cases are less common, less likely to have isolated skin lesions and bone involvement, and have higher rates of reactivation.^3^^,^^4^

Half of all LCH cases present with cutaneous findings. The classic morphology is an erythematous, papular rash on the groin, back, abdomen, and chest, along with yellow, raised, scaly papules on the scalp, raising suspicion of seborrheic dermatitis, intertrigo, hidradenitis suppurativa, or cutaneous Crohn’s disease.^5^^,^^6^ Our patient exhibited both reddish-brown ulcerated plaques with satellite papules in the groin, an atypical morphology for more common conditions like hidradenitis suppurativa or cutaneous Crohn’s disease, and therefore was not treated for such conditions. Immunohistochemical staining classically shows positivity for CD1a, Langerin (CD207), S100, and CD68, and genetic analysis shows a positive BRAF mutation in up to 70 percent of cases^1^; however, our case was BRAF and CD68-negative. The absence of positive CD68 markers should not rule out LCH if key diagnostic markers, CD1a, S100, and CD207, are positive. On electron microscopy, identification of Birbeck granules can further support a LCH diagnosis.

## Conflicts of interest

None disclosed.
